# Alternative duty work as workplace-initiated procedure to reduce sickness absence

**DOI:** 10.1186/s12889-021-11181-1

**Published:** 2021-06-16

**Authors:** Pauliina Mattila-Holappa, Johanna Kausto, Ville Aalto, Leena Kaila-Kangas, Mika Kivimäki, Tuula Oksanen, Jenni Ervasti

**Affiliations:** 1 Finnish Institute of Occupational Health, Helsinki, Finland; 2 Clinicum, Faculty of Medicine, University of Helsinki, Helsinki, Finland; 3 Department of Epidemiology and Public Health, University College London, London, UK; 4 Institute of Public Health and Clinical Nutrition, University of Eastern Finland, Kuopio, Finland

**Keywords:** Sickness absence, Work disability, Alternative duty work, Work modification

## Abstract

**Purpose:**

Alternative duty work is a procedure that enables an employee with a short-term disability to perform modified duties as an alternative to sickness absence. We examined whether the implementation of an alternative duty policy was associated with reduced sickness absence in the Finnish public sector.

**Methods:**

Two city administrations (A and D) that implemented an alternative duty work policy to their employees (*n* = 5341 and *n* = 7538) served as our intervention cities, and two city administrations (B and C) that did not implement the policy represented the reference cities (*n* = 6976 and *n* = 6720). The outcomes were the number of annual days, all episodes, and short-term (< 10 days) episodes during the 2 years before versus the 2 years after the intervention year. We applied repeated measures negative binomial regression analyses, using the generalized estimating equations method and the difference-in-difference analysis to compare the intervention and control cities (adjusted for sex, age, type of job contract, occupational class).

**Results:**

During the five-year study period, the number of sickness absence days and episodes increased in both the intervention and control cities. Covariate-adjusted analysis of relative risk showed that the overall increase in post- versus pre-intervention sickness absence days was smaller in intervention City A, RR = 1.14 (95% CI = 1.09–1.21) than in control cities B and C, RR = 1.19 (95% CI =1.14–1.24), group × time interaction *p* < 0.02. In intervention City D, we found a corresponding result regarding all sickness absence episodes and short-term sickness absence episodes but not days.

**Conclusions:**

This follow-up suggests that implementing an alternative duty work policy may marginally decrease employees’ sickness absences.

**Supplementary Information:**

The online version contains supplementary material available at 10.1186/s12889-021-11181-1.

## Introduction

Sickness absence (SA) is one of the major contributors to costs arising from work disability [1]. Prolonged SA is linked to a higher risk of disability pension [2–4] and unfavourable prognosis of return to work [5, 6]. Such SA may also contribute to poorer professional efficacy and competence, and the negative development of work-related self-efficacy [7, 8]. Return to work and remaining employed is a multifactorial process, with medical, psychological, social, and contextual aspects. Ability to work is not defined by only health or health impairment. Personal factors, self-evaluation of one’s functional capacity, workplace measures to support work ability, and social insurance systems also play a role [7, 9, 10]. Furthermore, as work ability may be defined in relation to either the demands of specific work tasks or work in general, the onset of illness, disability, or handicap may not necessarily lead to work disability [11].

The Finnish public sector, for example, has a number of procedures that aim to support work ability and prevent SA. The responsibilities of the employer to promote safety and health at work are defined in the Occupational Safety and Health Act [12]. An employer must identify and recognize the health and safety risks posed by work tasks, the work environment, and working conditions. The Finnish Occupational Health Care Act [13] also states that the employer has a duty to arrange occupational health services (OHS) for all their wage earners. In practice, OHS play a strong role in enhancing health at the workplace and public sector employers predominantly apply a model of early and active support, in which the employer provides support for employees with early signs of decreasing work ability [14]. Policy-level measures to prevent SA and disability retirement include legislation on part-time SA and the employer’s obligation to report prolonged SA to OHS. Longitudinal studies have found part-time SA to be associated with a smaller decline in labour market participation than full-time SA [15–19] and the practice of reporting prolonged SA cases to OHS has been linked to increased rates of continuing to work despite illness [20]. In addition, a recent study of public sector employees showed that the use of ‘return-to-work’ coordinators, while increasing SA, significantly reduced disability retirement [21].

Alternative duty work is a newly developed workplace policy and is applied in the Finnish public sector when an employee with a disability, illness or handicap is unable to return to their normal duties but is still able to perform modified duties or tasks at work [22]. The alternative duty work must comply with the person’s medical restrictions and not compromise their health or recovery. Some evidence shows that workplace interventions might reduce the time taken to return to work after SA [23] and that work modifications may decrease the duration of SA and speed up return to work [24–29]. The alternative duty work policy in Finland shares common features with some other policy-level measures to identify alternatives to SA, such as the sickness insurance programme in Norway [30], the Fit note policy in the UK [31] and alternative duties certificates in Australia [32, 33].

In Finland, alternative duty work is meant for individuals with short-term reduction of work ability as an alternative to SA [22]. However, it is unclear whether alternative duties are associated with future SA in this context.

Thus, we examined SA trends before and after the implementation of an alternative duty work procedure in municipalities using Finnish Public Sector Study (FPS) [34], which is a large cohort of public sector employees in Finland. The employees of the two municipalities that had implemented the alternative duty work procedure served as the intervention groups. The interventions took place irrespective of this study, which means that the researchers did not select the intervention group participants. The municipalities in which the procedure was not implemented served as controls. We excluded municipalities from the study if they implemented the procedure during the follow-up: participation in the intervention did not fulfil the criteria for controls, and starting the intervention late reduced the follow-up period, meaning that the criteria for participating in the intervention group were not met. The aim of this study was to determine whether the implementation of an alternative duty work policy was associated with

- the trend in annual SA days,
- the trend in the number of annual SA episodes, and.
- the trend in number of annual short-term (< 10 days) SA episodes.

## Materials and methods

**Fig. 1:**
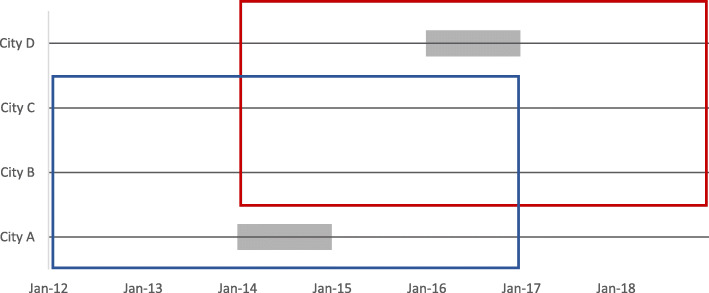
Participating cities and follow-up periods. Blue box indicates study wave 1: cases (City **A**) and controls (**B**-**C**) with sickness absence follow-up from 1.1.2012 (T1) to 31.12.2016 (T2). Red box indicates study wave 2: cases (City **D**) and controls (**B**-**C**) with sickness absence follow-up from 1.1.2014 (T1) to 31.12.2018 (T2). Grey bar indicates the wash-out period for the intervention (=the year of implementation of alternative duty work procedure)

We extracted the data from the FPS study of employees in the service of 11 municipalities across Finland [34]. In seven municipalities interviews [35] on employers’ work disability prevention practices in general, and on the alternative duty work method, were conducted. Of the seven municipalities, four were eligible for and were included in the present study on the associations between the alternative duty work procedure and SA. By taking advantage of a naturally occurring situation of implementing alternative duty work method in participating organizations, this study is a natural experiment nested in an observational cohort study. We compared SA trends 2 years before and 2 years after the implementation of the alternative duty work policy in the intervention and control cities. City A had implemented the method in 2014, and City D in 2016. Cities B and C had not implemented it and they were control cities. Figure 1 shows the study design.

### The intervention

Alternative duty work is a workplace policy and is applied in the Finnish public sector when an employee with a disability, illness or handicap is unable to return to their normal duties but is still able to perform modified duties or tasks at work. The process is most suitable for situations with short time decline of work ability [22]. The alternative duty work procedure was very similar in intervention Cities A and D. When an employee’s illness, disability, or injury continued after the self-certified SA period (maximum of 3–5 days), the employee visited an occupational health physician, who was responsible for evaluating the need of additional SA days and the applicability of alternative duty work. The supervisor decided on the alternative duties and, possibly together with a work ability coordinator, identified suitable work tasks, i.e. modified the employee’s tasks or duties. The work was not allowed to endanger recovery from illness, injury, or disability. Alternative duties could also include training or learning new skills [22]. Employees in alternative duty work were paid the same salary as in their normal duties. As employees in the public sector are paid a normal salary for the first 60 days of illness, there were no specific financial incentives to participate in alternative duty work. Participating in and organizing alternative duty work was voluntary. If the employee was unable to return to their duties after the period of alternative duty work, other interventions to support work ability were possible, such as partial SA and long-term work modifications [36].

### Study context

The public sector is a significant employer and branch of industry in Finland, engaging currently nearly half a million employees. In Finland, all non-retired residents aged 16 to 67 are eligible for a compensation of absence from work due to own illness. After the employment has lasted 1 month, the employee with a new work disability due to illness is eligible for receiving salary on the day on which the illness begins plus the following nine weekdays, paid by the employer. According to collective labour unions’ negotiated agreements, many employers continue paying full salary for the first one to 3 months of the work disability. In the public sector, full salary is paid for 2 months. After this, the Social Insurance Institution of Finland starts paying statutory sickness allowance, which compensates partly for lost wage income. Employers are obligated to inform OHS when an employee has been absent from work for 30 calendar days. When sick leave has lasted for 60 days, the employer must apply for sickness allowance from the Social Insurance Institution of Finland and the occupational health physician must evaluate the rehabilitation needs of the employee. When sickness allowance is been paid for a total of 90 days, OHS evaluates the work ability, and negotiates about the options of return to work with the employee and the employer. The maximum length of SA allowance is 300 working days per disease in 2 years. In case of long-term work disability, a full-time or part-time disability pension can be granted either temporarily or permanently [37, 38].

### Study population

The participants were employed by Cities A–D during the entire follow-up period for at least 6 months per year and were aged 18–68 years. Participants who were employed by the two participating municipalities during follow-up were omitted (4.2% of person-observations for City A and 1.9% for City D). This resulted in 5341 cases (City A) with 6976 controls, and 7538 cases (City D) with 6720 controls.

### Measures

The SA outcomes were defined as 1) the annual total number of SA days, 2) the annual total number of SA episodes, and 3) the annual total number of short-term (< 10 days) SA episodes during the 2 years before the intervention and during the 2 years after the intervention, as specified in Fig. 1. The SA data were retrieved from the employers’ registers and thus covered all episodes and days (from the first day onwards) during follow-up.

The covariates were sex (men/women), age, type of job contract (permanent/temporary/other), and occupational class [39], retrieved form the employers’ registers. Occupational class was based on the International Standard Classification of Occupations [40] of Statistics Finland. This classification has ten hierarchically ordered occupational classes: (1) legislators, senior officers and managers; (2) professionals; (3) technicians and associate professionals; (4) clerks; (5) service workers; (6) skilled agricultural and fishery workers; (7) craft and related trades workers; (8) plant and machine operators and assemblers; and (9) elementary occupations; and (10) armed forces (not included in the present data).

### Statistical analysis

We calculated the means of the annual SA days, SA episodes and short-term (< 10 days) SA episodes for each group during the five-year observation window, that is, 2 years before and 2 years after the year that the alternative duty work policy was implemented (intervention year being the wash-out year between the two-year periods).

To determine the association between the intervention and SA, we used difference-in-difference (DID) analysis. DID analysis determines the difference between post- and pre-intervention periods of two groups (intervention and control). This approach controls for non-measurable individual-level characteristics and common trends affecting both the intervention and control groups. We applied repeated measures negative binomial regression analyses using the generalized estimating equations (GEE) method with an exchangeable correlation structure and a logarithm function of annual person-months in employment as the offset-variable. This variable accounts for time at risk of SA. The repeated-measures GEE method considers intraindividual correlation between the measurements, and results in rate ratio estimates of the risk after versus before the intervention, with 95% confidence intervals (CI). To determine whether the change in time was different across the intervention and control cities, we entered the interaction term ‘group × year’ into the model. Year was specified as a class variable in the analysis. To determine whether the common trends assumption in the DID analysis was met [41, 42], we tested the interaction term ‘group × years before the intervention’. The assumption was met for City A vs. controls for SA days, and for City D vs. controls for all outcomes. Thus, we did not analyse SA episodes for City A but only for City D compared to control Cities B and C.

All analyses were performed using SAS statistical software, version 9.4 [43].

## Results

Table 1 presents the descriptive statistics of the participants by intervention status. The participants in the control Cities B and C were more often women than those in intervention Cities A and D. In intervention cities A and D, fewer participants had an employment contract classified as ‘other’ (contract not classified as permanent or temporary) than in the control cities. Intervention City D had more managers and professionals than the control cities.

**Table 1:** Descriptive characteristics of participants by case/control status at start of follow-up. Frequency (percentage) or Mean (SD)

	City A: Alternative duty work in use in 2014	Control Cities		City D: Alternative duty work in use in 2016	Control Cities	
	Case 43% (*n* = 5341)	Control 57% (*n*= 6976)	P for difference	Case 53% (*n*= 7538)	Control 47% (*n*= 6720)	P for difference
**Sex**: Men	1486 (28)	1633 (23)		2299 (31)	1530 (23)	
Women	3855 (72)	5343 (77)	< 0.001	5239 (69)	5190 (77)	< 0.001
**Employment contract**						
Permanent	4485 (84)	5746 (82)		6650 (88)	5600 (83)	
Temporary	833 (16)	1161 (17)		866 (11)	1069 (16)	
Other	23 (0.4)	69 (1)	< 0.001	22 (0.3)	51 (1)	< 0.001
**Occupational class **						
Elementary workers	259 (5)	371 (5)		461 (6)	337 (5)	
Skilled manual	1735 (33)	2257 (33)		2454 (33)	2127 (32)	
Lower grade non-manual	1338 (25)	1640 (24)		1438 (19)	1504 (23)	
Managers and professionals	2000 (37)	2661 (38)	0.20	3160 (42)	2673 (40)	< 0.001
**Age:** mean (SD)	45.9 (8.8)	46.0 (9.3)		45.6 (9.3)	46.0 (9.4)	

**Fig. 2:**
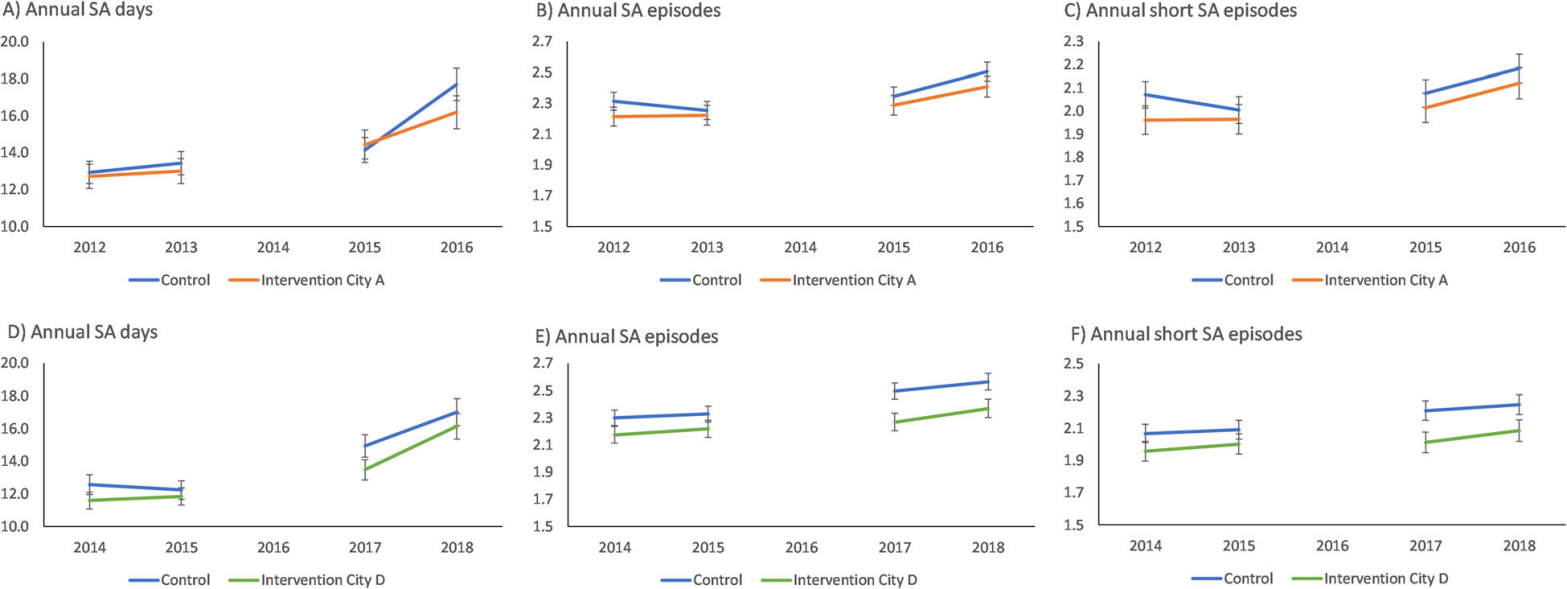
Unadjusted (observed) means of days and episodes of sickness absence (SA) per year before and after intervention in intervention cities and controls. Panels **A**, **B**, and **C** show trends annual SA days, annual SA episodes, and annual short SA episodes in intervention city A compared to controls. Panels **D**, **E**, and **F** show respective trends in intervention city D compared to controls

Figure 2 shows the unadjusted (observed) means of days and episodes of sickness absence (SA) per year before and after intervention in intervention cities and controls. Panels A, B, and C show trends, annual SA days, annual SA episodes, and annual short SA episodes in intervention city A in comparison to controls. Panels D, E, and F show the respective trends in intervention city D in comparison to controls.

Table 2 shows the risk ratio for the annual number of SA days, annual number of SA episodes, and annual number of short-term SA episodes after the alternative duty work method versus before it in the intervention and control cities, as well as the *p*-values for the ‘group × time’ interaction term.

**Table 2:** Ratio of annual days of sickness absence (SA), annual number of SA episodes, and annual number of short-term (< 10 days) SA episodes after intervention versus before intervention compared to controls. Years 1 and 2 after the intervention are contrasted to years −2 and − 1 before the intervention. Models are adjusted for sex, age, employment contract and occupation

	City A: Alternative duty work in use in 2014		Control Cities			City D: Alternative duty work in use in 2016		Control Cities		
	RR	95% CI	RR	95% CI	Group*time interaction^a^	RR	95% CI	RR	95% CI	Group*time interaction^a^
**SA days**										
2 years before	1		1			1		1		
2 years after	1.14	1.09–1.21	1.19	1.14–1.24	0.02	1.21	1.15–1.26	1.25	1.19–1.31	0.12
**SA episodes**										
2 years before						1		1		
2 years after						1.08	1.06–1.10	1.13	1.11–1.15	< 0.001
**Short SA episodes**										
2 years before						1		1		
2 years after						1.07	1.05–1.09	1.12	1.10–1.14	< 0.001

^a^Group*time interaction (*p*-value)

The mean number of SA days per year was similar during the pre-intervention years in intervention City A and in the control municipalities (13 days per year). One year after the implementation of the alternative duty work policy in 2014, the mean number of SA days was higher in both the intervention and control cities (14 days). The mean number of SA days greatly increased during the second post-intervention year among the controls (18 days), but not as much in the intervention City A (16 days) (Fig. 2, panel A). In the covariate-adjusted (sex, age, employment contract and occupational class) analysis of relative risk (DID) shown in Table 2, a higher risk of SA days 2 years before the intervention compared to 2 years after the intervention was observed in both the intervention and control cities. In intervention City A, the relative risk of SA days after the interventions was 1.14 (95% CI 1.09–1.21) in comparison to before the intervention. However, the corresponding increase among the controls was higher, RR = 1.19 (95% CI 1.14–1.24), as confirmed by a significant group × time interaction between intervention City A and the controls, *p* = 0.02. The (increasing) trend in annual SA days in intervention City D and the controls was similar (Fig. 2, panel D; Table 2).

In intervention City D, the mean number of SA episodes during the 2 years before the intervention (2014–2015) was 2.2. The corresponding mean number of SA episodes in the control cities was slightly higher, at 2.3 episodes. After the intervention (2017–2018), the mean number of SA episodes was 2.3–2.4 for intervention City D, and 2.5–2.6 for the controls. (Fig. 2, panel E.) The covariate-adjusted (sex, age, employment contract and occupational class) analysis of relative risk (Table 2) confirmed that the relative risk of SA episodes increased in both the intervention and control cities, but slightly more in the control cities. In intervention City D, the relative risk of SA episodes after the intervention was 1.08 (95% CI 1.06–1.10) in comparison to before the intervention. However, the corresponding increase in risk among the controls was higher (1.13; 95% CI 1.11–1.15), as confirmed by a significant group × time interaction (*p* < 0.001). The trend in mean number of SA episodes before the intervention differed in City A and the controls (*p* = 0.03; Fig. 2, panel B). Thus, Table 2 does not report the DID analysis.

The pre-intervention mean of short-term (< 10 days) SA episodes was slightly higher among the controls (2.1 episodes per year) than in intervention City D (2.0 episodes). Two years after the intervention (2017–2018), the mean in City D was from 2.0 to 2.1. The corresponding means of the controls were 2.2–2.3. Thus, from pre- to post-intervention, there was an increase in both the intervention Cities and the controls, but the increase was greater among the controls. (Fig. 2, panel F). The covariate-adjusted analysis (sex, age, employment contract and occupational class) of relative risk (Table 2) confirmed this result: In City D, the RR for short-term SA after the intervention was 1.07 (95% CI = 1.05–1.09) in comparison to before the intervention. Among the controls, the increase was higher (RR = 1.12, 95% CI = 1.10–1.14), as confirmed by a significant group × time interaction (*p* < 0.001). The short-term SA episode trend before the intervention differed in City A and the controls (*p* = 0.02; Fig. 2, panel C). Thus, Table 2 does not report the DID analysis.

## Discussion

We studied whether the introduction of an alternative duty work policy in two Finnish municipalities was associated with changes in employee SA. We found that the annual number of SA days and SA episodes increased in both the intervention and control cities. However, the increase in SA days and episodes was smaller in the intervention cities than in the control cities, although the results were not uniform in both intervention cities. In one intervention city, the annual SA days increased less than in the control cities, and in another intervention city, the annual SA episodes increased less than in the control cities. The arrangement and processes of OHS were very similar in the participating cities. However, the different effects of alternative work duty policy may reflect variation in the practices by which SA is issued.

Previous studies have shown that work modification measures [24–29] and gradual and tailored return to work [44] may increase work participation despite illness. In Finland, alternative duty work may complement the earlier effective policy-level measures that have been used in the Finnish public sector to prevent and shorten SA, including the partial sickness absence policy [15–19], the policy that obligates employers to report prolonged SA and OHS to contact the employee and evaluate work ability during SA [20], and the use of ‘return-to-work’ coordinators [21], It has been suggested that combining different measures to support work ability is particularly beneficial [26, 45, 46]. Alternative duty work might serve as an important part of measures to support work ability.

Several earlier studies have investigated policies that aim to reduce SA and support employees with health conditions. Markussen et al. [30] for example, reported favourable outcomes from a sickness insurance programme in Norway. The absentees who were assigned graded (partial) absence certificates had higher subsequent employment rates than those on regular sick leave. This procedure did not include a change of duties and shared similarities with the Finnish partial sickness absence policy [15].

In some previous studies, the simultaneous use of multiple explanatory measures has made it difficult to determine specific effects. For example, the study on the effects of the Danish return-to-work programme on long-term SA showed that the results of the municipalities differed significantly, and that adjustment for individual-level confounding factors only had a small impact on the estimates [47]. The authors concluded that the variation of the unmeasured contextual factors in the municipalities may have confounded the results.

The effect of the intervention may also differ by subgroup. The Fit Note policy in the UK has reduced long-term SA episodes in general, but an increase in mental health-related SA has also been observed [31]. A five-year follow-up study in Norway, the Inclusive Working Life programme, observed no effects on the populations overall, but long-term SA decreased among male shift workers [48]. In Australia, ‘alternative duties’ certificates have been an alternative to ‘unfit for work’ certificates, and their use remained at a stable level between 2003 and 2010, although illness categories differed [32, 33].

From an employee perspective, alternative duties, as an alternative to SA, may enable receiving support from the work community without risking health or recovery. However, the system has been criticized for increasing the employer’s decision-making authority in health-related matters, and employers sometimes have difficulties finding motivating duties for employees. Alternative duty work is nevertheless based on negotiations between the employee and employer, requires the consent of the employee, and obliges OHS to ensure that the alternative duties do not harm the health or recovery of the employee. To identify suitable alternative duties, the supervisor must understand the relationship between the demands of the work and health and make use of OHS’ expertise. In the current system, employees with alternative duties work full time. Depending on the nature of the employee’s illness or injury, in some cases partial sickness benefit may be a more suitable option for supporting future work ability.

Currently, alternative work is not part of the public sector collective agreement, but many public sector employers have implemented the policy, including the municipalities in the current study. Alternative duty work may be easier to implement in larger organizations, whereas the limited number of different tasks may restrict possibilities in smaller organizations. Employees doing alternative duty work are paid the same salary as that for their normal duties. Thus, as employees in the public sector are paid a normal salary for the first 60 days of illness, alternative duty work offers no specific financial incentives [37].

The strengths of this study were the large cohort data with several years’ follow-up, the participation of multiple cities, and the comprehensive register data on SA from committed public employers. Our study includes also some limitations. The SA data did not include the diagnoses due to which the alternative duties were implemented. The statistics of the intervention cities showed that approximately 1–4% of the personnel had worked at least 1 day of alternative duties per year, after the procedure had been introduced. Participation in alternative duties is likely to be affected by selection bias, as participating in alternative duties is voluntary. However, according to the experiences in the intervention cities, most of the employees to whom alternative duties were recommended wanted to participate.

The difference in difference analysis includes the assumption that in the absence of the intervention, the outcomes would have changed in the same way in both the intervention and control cities, and that the unobserved characteristics would have been fixed [42]. These assumptions are difficult to test. The method was nevertheless chosen, as the participating cities were similar in terms of population, prevalence of SA, and operational environment. The results may be generalized to the public sector, but whether they can be applied to other sectors, or to other countries with differing social security policies, is not sure.

In the coming years, economies throughout the Western world will be challenged by demographic change and a shrinking working-age population [49]*.* Implementing alternative duty work may influence SA in at least in two ways, and the benefits may be either short- or long-term. First, when an employee with health problems can work doing alternative duties, SA as such may be avoided. Second, performing alternative duties may prevent the known negative effects of long SA on general health, including a deterioration of work ability [5, 6] or a worsening self-evaluation of it [7, 8]. It has been shown that a combination of different measures to support work ability might be the most beneficial [23, 26, 45, 46]. Alternative duty work should thus be combined with other measures that have proven to be effective.

## Conclusions

In conclusion, our results from Finnish public sector workplaces suggest that implementing an alternative duty work policy in cases of temporary decline in work ability can result in less SA among the employees. If these findings are successfully replicated across different settings, alternative duty work can be recommended as a procedure to reduce SAs.

## Supplementary Information

**Additional file 1.** Interview guide on employers’ work disability prevention practices in general, and on the alternative duty work method.

## Data Availability

The datasets analyzed during the study are not publicly available due to sensitive and health-related nature of the data but anonymized data are available from the corresponding author on reasonable request.

## Abbreviations

CI: Confidence Interval
DID: Difference in Difference
FPS: Finnish Public Sector Study
GEE: Generalized Estimating Eqs.
RR: Relative Risk
SA: Sickness Absence
SD: Standard Deviation

## References

[1] OECD (2020). Sickness, disability and work: Breaking the barriers.

[2] Laaksonen M, Blomgren J, Gould R (2016). Sickness allowance trajectories preceding disability retirement: a register-based retrospective study. Eur J Pub Health.

[3] Laaksonen M, Blomgren J (2016). Tuulio-Henriksson a sickness allowance histories among disability retirees due to mental disorders: a retrospective case-control study. Scand J Public Health.

[4] Laaksonen M, He L, Pitkaniemi J (2013). The durations of past sickness absences predict future absence episodes. J Occup Environ Med.

[5] Virtanen M, Kivimäki M, Vahtera J (2016). Sickness absence as a risk factor for job termination, unemployment, and disability pension among permanent and temporary employees. Occup Environ Med.

[6] Vahtera J, Westerlund H, Ferrie JE, Head J, Melchior M, Singh-Manoux A, Zins M, Goldberg M, Alexanderson K, Kivimaki M (2010). All-cause and diagnosis-specific sickness absence as a predictor of sustained suboptimal health. A 14-year follow-up in the GAZEL cohort. J Epidemiol Community Health.

[7] Blank L, Peters J, Pickvance (2008). A systematic review of factors which predict return to work for people suffering episodes of poor mental health. J Occup Rehabil.

[8] Volker D, Zijlstra-Vlasveld MC, Brouwers EP (2015). Return-to-work self-efficacy and actual return to work among long-term sick-listed employees. J Occup Rehabil.

[9] Lederer V, Loisel P, Rivard M, Champagne F (2014). Exploring the diversity of conceptualizations of work (dis)ability: a scoping review of published definitions. J Occup Rehabil.

[10] de Wind A, Geuskens GA, Ybema JF (2014). Job characteristics, skills, and social and financial factors in relation to early retirement-results from a longitudinal study in the Netherlands. Scand J Work Environ Health.

[11] Tengland PA (2011). The concept of work ability. J Occup Rehabil.

[12] Occupational Safety and Health Act. http://www.finlex.fi/en/laki/kaannokset/2002/en20020738. Accessed 20 Apr 2021.

[13] Occupational Health Care Act. http://www.finlex.fi/en/laki/kaannokset/2001/en20011383. Accessed 20 Apr 2021.

[14] Model of early support. Keva 2015. https://www.keva.fi/globalassets/2-tiedostot/ta-tiedostot/tyoelamapalvelut/liite_aktiivisen_tuen_malli_tekme.pdf). Accessed 20 Apr 2021.

[15] Kausto J, Miranda H, Martimo KP, Viikari-Juntura E (2008). Partial sick leave - review of its use, effects and feasibility in the Nordic countries. Scand J Work Environ Health.

[16] Kausto J, Viikari-Juntura E, Virta LJ, Gould R, Koskinen A, Solovieva S (2014). Effectiveness of new legislation on partial sickness benefit on work participation: a quasi-experiment in Finland. BMJ Open.

[17] Shiri R, Kausto J, Martimo KP, Kaila-Kangas L (2013). Health-related effects of early part-time sick leave due to musculoskeletal disorders: a randomized controlled trial. Scand J Work Environ Health.

[18] Viikari-Juntura E, Virta LJ, Kausto J, Autti-RÃ¤mÃ I, Martimo KP, Laaksonen M, Leinonen T, Husgafvel-Pursiainen K, Burdorf A, Solovieva S (2017). Legislative change enabling use of early part-time sick leave enhanced return to work and work participation in Finland. Scand J Work Environ Health.

[19] Ervasti J, Kausto J, Koskinen A, Pentti J, Vahtera J, Joensuu M, Turunen J, Oksanen T, Kivimäki M (2020). Labour market participation before and after long-term part-time sickness absence in Finland: a population-based cohort study. J Occup Environ Med.

[20] Halonen J, Solovieva S, Virta LJ (2018). Sustaining return to work and work participation after new legislation obligating employers to notify prolonged sickness absence. Scand J Public Health.

[21] Kausto J, Oksanen T, Koskinen A, Pentti J, Mattila-Holappa P, Leena Kaila-Kangas L, Nevala N, Kivimäki M, Vahtera J, Ervasti J (2021). Return to work’ coordinator model and work participation of employees: a natural intervention study in Finland.

[22] Pekkarinen L, Haapakoski S. Alternative duties in municipal sector. Planning and implementation. Keva. Helsinki; 2017.

[23] van Vilsteren M, van Oostrom SH, de Vet HC (2015). Workplace interventions to prevent work disability in workers on sick leave. Cochrane Database Syst Rev.

[24] Andersen MF, Nielsen KM, Brinkmann S (2012). Meta-synthesis of qualitative research on return to work among employees with common mental disorders. Scand J Work Environ Health.

[25] Bastien M-F, Corbiere M (2019). Return-to-work following depression. What work accommodations do employers and human resources directors put in place. J Occup Rehabil.

[26] Cullen KL, Collie A, Clay F (2018). Effectiveness of workplace interventions in return-to-work for musculoskeletal, pain-related and mental health conditions: an update of the evidence and messages for practitioners. J Occup Rehabil.

[27] Franche R-L, Cullen K, Clarke J, Irvin E, Sinclair S, Frank J, The Institute for Work & Health (IWH) Workplace-Based RTW Intervention Literature Review Research Team (2005). Workplace-based return to work interventions: a systematic review of the quantitative literature. J Occup Rehabil.

[28] Nevala N, Pehkonen I, Koskela I (2014). Workplace accommodation among persons with disabilities. A systematic review on it’s effectiveness and barriers of facilitators. J Occup Rehabil.

[29] Johansson G, Lundberg O, Lundberg I (2006). Return to work and adjustment latitude among employees on long-term sickness absence. J Occup Rehabil.

[30] Markussen S, Mykletun A, Røed K (2012). The case for presenteeism-Evidence from Norway’s sickness insurance program. J Public Econ.

[31] Gabbay M, Shields C, Hillage J (2015). Factors associated with the lengt of fit-note certified sickness episodes in the UK. Occup Environ Med.

[32] Ruseckaite R, Collie A, Maatje Scheepers M (2016). Factors Associated With Sickness Certification of Injured Workers by General Practitioners in Victoria, Australia. BMC Public Health.

[33] Collie A, Ruseckaite R, Brijnath B (2016). Sickness certification of workers compensation claimants by general practitioners in Victoria, 2003-2010. Med J Aust.

[34] Ervasti J, Airaksinen J, Pentti J, Vahtera J, Suominen S, Virtanen M, Kivimäki M (2019). Does increasing physical activity reduce the excess risk of work disability among overweight individuals?. Scand J Work Environ Health.

[35] Interview guide on employers’ work disability prevention practices in general, and on the alternative duty work method. Interview guide developed for the current study. Included as supplementary material.

[36] Model of early support. Keva 2015. https://www.keva.fi/globalassets/2-tiedostot/ta-tiedostot/tyoelamapalvelut/liite_aktiivisen_tuen_malli_tekme.pdf

[37] Public Sector collective agreement 2020. https://www.kt.fi/sopimukset/kvtes/2020-2021/luku-5-virka-ja-tyovapaat-seka-perhevapaat/sairausloma, https://www.kela.fi/sairauspaivaraha. Accessed 20 Apr 2021.

[38] Sickness benefit. Kela 2020. https://www.kela.fi/sairauspaivaraha. Accessed 20 Apr 2021.

[39] Kivimäki M, Head J, Ferrie JE, Shipley MJ, Vahtera J, Marmot MG (2003). Sickness absence as a global measure of health: evidence from mortality in the Whitehall II prospective cohort study. BMJ.

[40] International Standard Classification of Occupations (ISCO) of Statistics Finland. http://www.stat.fi/meta/luokitukset/ammatti/001-2001/kuvaus_en.html. Accessed 24 Sept 2020.

[41] Craig P, Cooper C, Gunnell D, Haw S, Lawson K, Macintyre S, Ogilvie D, Petticrew M, Reeves B, Sutton M, Thompson S (2012). Using natural experiments to evaluate population health interventions: new Medical Research Council guidance. J Epidemiol Community Health.

[42] Dimick JB, Ryan AM (2014). Methods for evaluating changes in health care policy: the difference-in-differences approach. JAMA.

[43] SAS statistical software, version 9.4 (SAS Institute, Cary, NC).

[44] Lagerveld S, Houtman I. Return to work after sick leave due to mental health problems. OSHWiki 2017. https://oshwiki.eu/wiki/Return_to_Work_after_sick_leave_due_to_mental_health_problems. Accessed 6 May 2020.

[45] Pomaki G, Franche R, Murray E, Khushrushahi N, Lampinen TM (2012). Workplace-based work disability prevention interventions for workers with common mental health conditions: review of the literature. J Occup Rehabil.

[46] Mikkelsen M, Rosholm M (2018). Systematic review and meta-analysis of interventions aimed at enhancing return to work for sick-listed workers with common mental disorders, stress-related disorders, somatoform disorders and personality disorders. Occup Environ Med.

[47] Poulsen OM, Aust B, Bjorner JB, Rugulies R, Hansen JV, Tverborgvik T, Winzor G, Mortensen OS, Helverskov T, Ørbæk P, Nielsen MBD (2014). Effect of the Danish return-to-work program on long-term sickness absence: results from a randomized controlled trial in three municipalities. Scand J Work Environ Health.

[48] Foss L, Gravseth HM, Kristensen P, Claussen B, Mehlum IS, Skyberg K (2013). Inclusive working life in Norway: a registry-based five-year follow-up study. J Occup Med Toxicol.

[49] European Commission report on the impact of demographic change. European Commission 2020. demography_report_2020_n.pdf (europa.eu). Accessed 8 Apr 2021.

