# Supplementary material for: Alternative duty work as workplace-initiated procedure to reduce sickness absence
Source: BMC Public Health. 2021 Jun 16;21:1154. doi: 10.1186/s12889-021-11181-1 (PMC8207644; doi:10.1186/s12889-021-11181-1)
Supplement: Supplementary file 1 — Additional file 1. Interview guide on employers’ work disability prevention practices in general, and on the alternative duty work method. [file 12889_2021_11181_MOESM1_ESM.pdf]

Interview guide: Work ability promotion in municipal sector

[Details of the interviewee, occupational position, work unit]

1. Which of the following options describes best the work ability model in your work unit? The model of active support for work ability is used
0 = not at all [interview ends]
1 = partly
2 = used, but not consistently
3 = used consistently

2. When was the model implemented? [date]

3. Which parties were involved in model development?
1 = top management
2 = HR
3 = supervisors / middle management
4 = occupational health care
5 = employees
6 = other, who

4. Are the roles and responsibilities in work ability promotion clear?

1 = totally unclear
2 = somewhat unclear
3 = not clear, but not unclear
4 = rather clear
5 = totally clear

5. Are the resources (budget, staff) assigned for work ability promotion sufficient?

1 = totally sufficient
2 = rather sufficient
3 = insufficient in my organization
4 = insufficient in occupational health care
5 = don’t know / don’t want to evaluate
6 = other

EARLY SUPPORT FOR WORK ABILITY PROMOTION

6. Is the model for early support for work ability promotion at use in your work unit?

1 = not at all [to Q15]
2 = partly
3 = yes, but not throughout the unit
4 = yes, consistently and throughout the unit

7. Which procedures are included

1 = sickness absence management
2 = short-term sickness absence, early intervention
3 = self-certification of sickness absence
4 = wellbeing and work ability training, career management
5 = work disability risk management
6 = work modification (tools, flexibility in workplace and time, work tasks)
7 = other, what

8. Were all procedures implemented simultaneously?

1 = yes [to Q9]
0 = no

8.1 Which procedures were implemented separately [procedure 1, date; procedure 2, date; procedure N, date]

9. When was the model of early support for work ability promotion implemented? [date]

10. Is the model of early support directed towards a specific group of employees?

0 = No [Q11]
1 = Yes

10.1 To which group?

11. Who is responsible for the implementation and management of model of early support for work ability?

1 = unit supervisor
2 = HR
3 = occupational health care
4 = other

12. In practice, how often is the model used?

0 = there is practically no need for it
1 = there is very little need for it
2 = inconsistently, when needed [Q12.1]
3 = systematically
4 = other

12.1. If the model is used inconsistently, where/in which cases it has been implemented and where/in which cases not?

13. Are supervisors trained to use model of early support for work ability?

0 = No [Q13.3]
1 = Yes [Q13.1]

13.1 When was the training? [date]

13.2 Was participation to the training active?

1 = practically every supervisor has participated
2 = participation has varied
3 = very few supervisors have participated

13.3 Have other means than training been used to disseminate information on the model?

0 = No
1 = Yes, how?

ACCELERATED SUPPORT FOR WORK ABILITY

14. Is the model for accelerated support for work ability at use in your work unit?

1 = not at all [to Q22]
2 = partly
3 = yes, but not throughout the unit
4 = yes, consistently and throughout the unit

15. Which procedures are included

1 = part-time work
2 = part-time work + supported pay
3 = partial sickness absence
4 = return-to-work coordinator, work ability manager
5 = rehabilitation manager
6 = occupational rehabilitation
7 = part-time work disability pension
8 = alternative duty work

9 = work try-out / work trial
10 = work modification/accommodation
11 = other, what

16. Were all procedures implemented simultaneously?

1 = yes [to Q17]
0 = no

16.1 Which procedures were implemented separately [procedure 1, date; procedure 2, date; procedure N, date]

17. When was the model of accelerated support for work ability implemented? [date]

18. Is the model of accelerated support for work ability directed towards a specific group of employees?

0 = No [Q20]
1 = Yes

18.1 To which group?

19. Who is responsible for the implementation and management of model of accelerated support for work ability?

1 = unit supervisor
2 = HR
3 = occupational health care
4 = other

20. In practice, how often is the model used?

0 = there is practically no need for it
1 = there is very little need for it
2 = inconsistently, when needed [Q20.1]
3 = systematically
4 = other

20.1. If the model is used inconsistently, where/in which cases it has been implemented and where/in which cases not?

21. Are supervisors trained to use model of accelerated support for work ability?

0 = No [Q21.3]
1 = Yes [Q21.1]

21.1 When was the training? [date]

21.2 Was participation to the training active?

1 = practically every supervisor has participated
2 = participation has varied
3 = very few supervisors have participated

21.3 Have other means than training been used to disseminate information on the model?

0 = No
1 = Yes, how?

SUPPORT FOR RETURN TO WORK

22. Is the model for support for return to work at use in your work unit?

1 = not at all [interview ends]
2 = partly
3 = yes, but not throughout the unit
4 = yes, consistently and throughout the unit

23. Which procedures are included

1 = occupational health negotiations
2 = work try-outs / trials
3 = partial sickness absence
4 = part-time work (+support pay)
5 = work modification / work accommodation
6 = reorientation
7 = supervisor-employee negotiations
8 = orientation of the work unit to returning employee / consultation of occupational psychologist

24. Were all procedures implemented simultaneously?

1 = yes [to Q25]
0 = no

24.1 Which procedures were implemented separately [procedure 1, date; procedure 2, date; procedure N, date]

25. When was the model of support for return to work implemented? [date]

26. Is the model of support for return to work directed towards a specific group of employees?

0 = No [Q27]
1 = Yes

26.1 To which group?

27. Who is responsible for the implementation and management of model of support for return to work?

1 = unit supervisor
2 = HR
3 = occupational health care
4 = other

28. In practice, how often is the model used?

0 = there is practically no need for it
1 = there is very little need for it
2 = inconsistently, when needed [Q28.1]
3 = systematically
4 = other

28.1. If the model is used inconsistently, where/in which cases it has been implemented and where/in which cases not?

29. Are supervisors trained to use model of support for return to work?

0 = No [Q29.3]
1 = Yes [Q13.1]

29.1 When was the training? [date]

29.2 Was participation to the training active?

1 = practically every supervisor has participated
2 = participation has varied
3 = very few supervisors have participated

29.3 Have other means than training been used to disseminate information on the model?

0 = No
1 = Yes, how?

30. How is the implementation of work ability promotion practices and procedures being followed?

31. Is there any printed/printable materials related to work ability promotion practices and procedures available?

32. Would you like to add something related to work ability promotion practices and procedures? [interview ends]
